# Splenosis with lower gastrointestinal bleeding mimicking colonical gastrointestinal stromal tumour

**DOI:** 10.1186/s12957-017-1153-0

**Published:** 2017-04-11

**Authors:** Shuo-meng Xiao, Rui Xu, Xiao-li Tang, Zhi Ding, Ji-man Li, Xiang Zhou

**Affiliations:** 1 Department of Gastrointestinal Surgery, Sichuan Cancer Hospital, No.55, South 4th Section, Renmin Road, Chengdu, Sichuan Province China; 2 Department of Pathology, Sichuan Cancer Hospital, Chengdu, China

**Keywords:** Splenosis, GIST, Nuclear scintigraphy, Ferumoxide-enhanced MRI, CT

## Abstract

**Background:**

Splenosis refers to the heterotopic transplantation of splenic tissue following splenic trauma or splenectomy. Splenosis is typically asymptomatic and is often identified incidentally.

**Case presentation:**

We report a case of splenosis with colon and stomach invasion presenting as lower gastrointestinal bleeding and mimicking colonic gastrointestinal stromal tumour (GIST). The importance of suspicion for splenosis in patients with a history of splenic injury should be highlighted. Computed tomography (CT)-guided biopsy, nuclear scintigraphy and ferumoxide-enhanced magnetic resonance imaging (MRI) can support an accurate diagnosis.

**Conclusions:**

An accurate diagnosis of splenosis is important to avoid unnecessary operations, especially in patients with previous histories of splenic trauma or splenectomy.

## Background

Splenosis was first described by Buchbinder and Lipkopf in 1939 and is reported to occur in nearly 65% of patients who experience splenic trauma and splenectomy [1]. Most patients with splenosis are asymptomatic. Cases of splenosis are usually misdiagnosed as tumours of the thorax, abdomen and pelvis, and patients often undergo unnecessary operations [2–5]. Here, we report a case of splenosis presenting with lower gastrointestinal bleeding indistinguishable from colonic gastrointestinal stromal tumour (GIST). Upper gastrointestinal endoscopy and colonoscopy were performed, and a mass was identified in the splenic flexure of the colon. CT imaging revealed that this mass invaded the colon and stomach. A clinical diagnosis of colon cancer or colonic GIST was made. Local colon excision and local stomach excision were performed. Pathological examination revealed this neoplasm to be splenosis.

## Case presentation

**Fig. 1:**
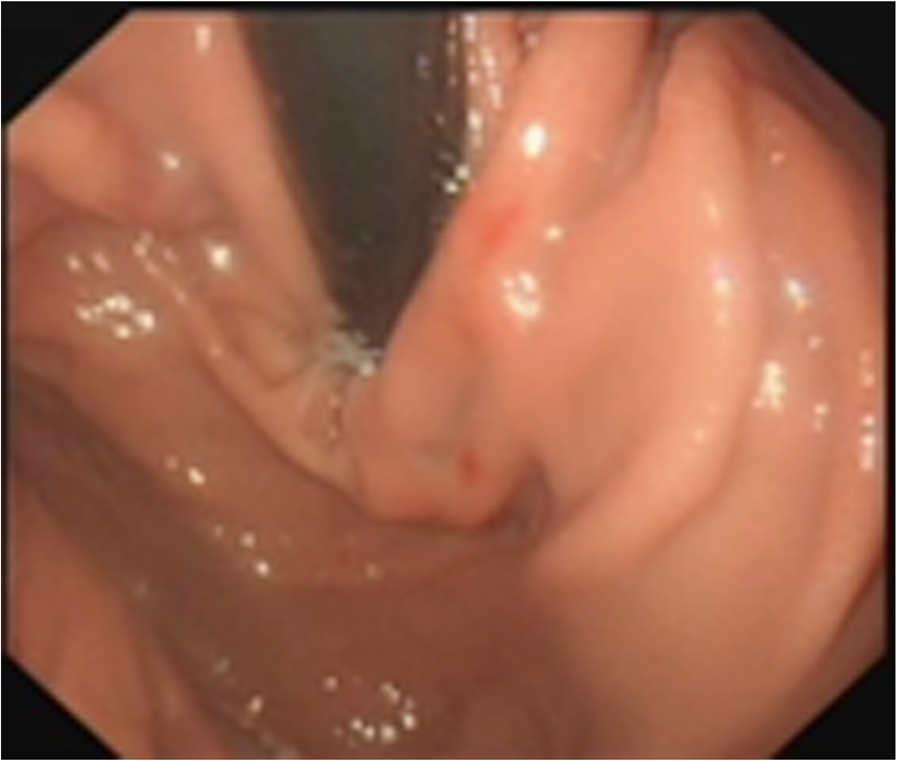
No mass was found by upper gastrointestinal endoscopy

**Fig. 2:**
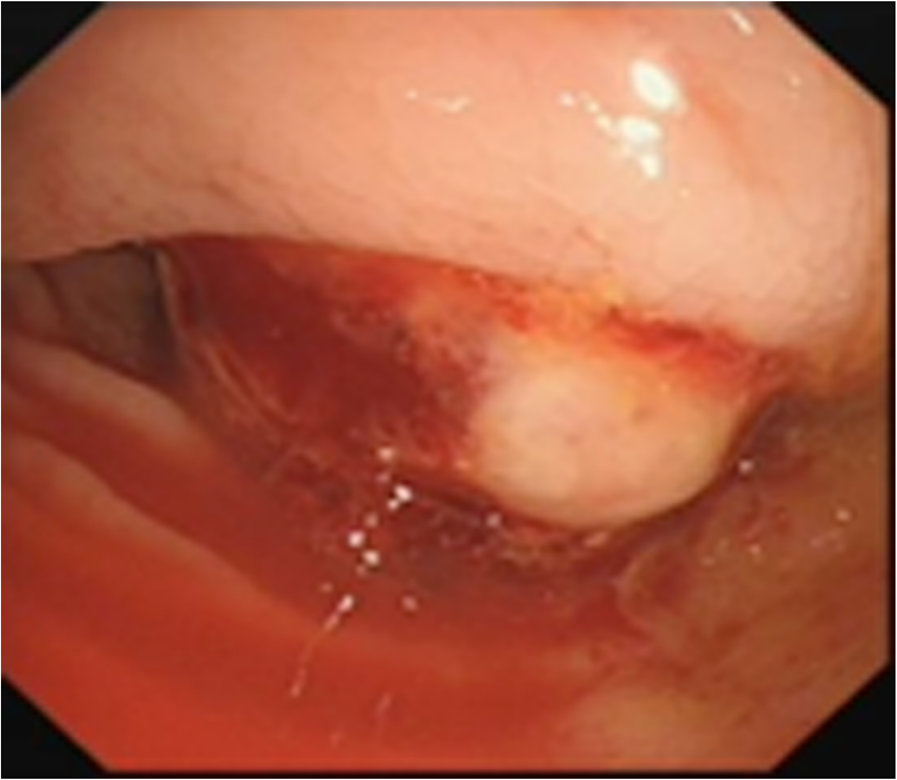
Colonoscopy revealed a large, round neoplasm in the splenic flexure of the colon

**Fig. 3:**
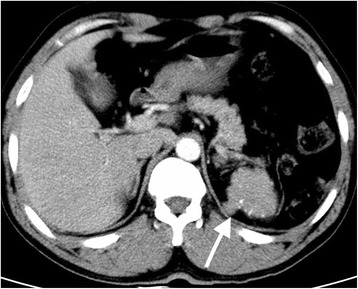
CT showing a huge tumour located in the splenic fossa and the tumour invading into the splenic flexure of the colon

The patient was a 40-year-old man who had undergone splenectomy secondary to a traffic accident 10 years prior to presentation. He reported recurrent melena for 1 month prior to admission. On admission, the patient’s vital signs were normal, and physical examination was unremarkable. Laboratory findings revealed a haemoglobin level of 44 g/L. After blood transfusion, the patient’s haemoglobin level increased to 80 g/L. All tumour marker values were normal. To identify the cause of his gastrointestinal bleeding, upper gastrointestinal endoscopy and colonoscopy were performed. Upper gastrointestinal endoscopy revealed chronic superficial gastritis, and no mass was found (Fig. 1). Colonoscopy revealed a large, round neoplasm in the splenic flexure of the colon that had the appearance of a colon cancer or colonic gastrointestinal stromal tumour (Fig. 2). Biopsy revealed chronic colitis, and no tumour cells were identified. An abdominal contrast-enhanced CT revealed a very large mass located in the splenic fossa that was approximately 5 cm in diameter and appeared to invade the splenic flexure of the colon (Fig. 3); the lesion was consistent with colon tumour. Thus, a clinical diagnosis of colonic GIST was made.

**Fig. 4:**
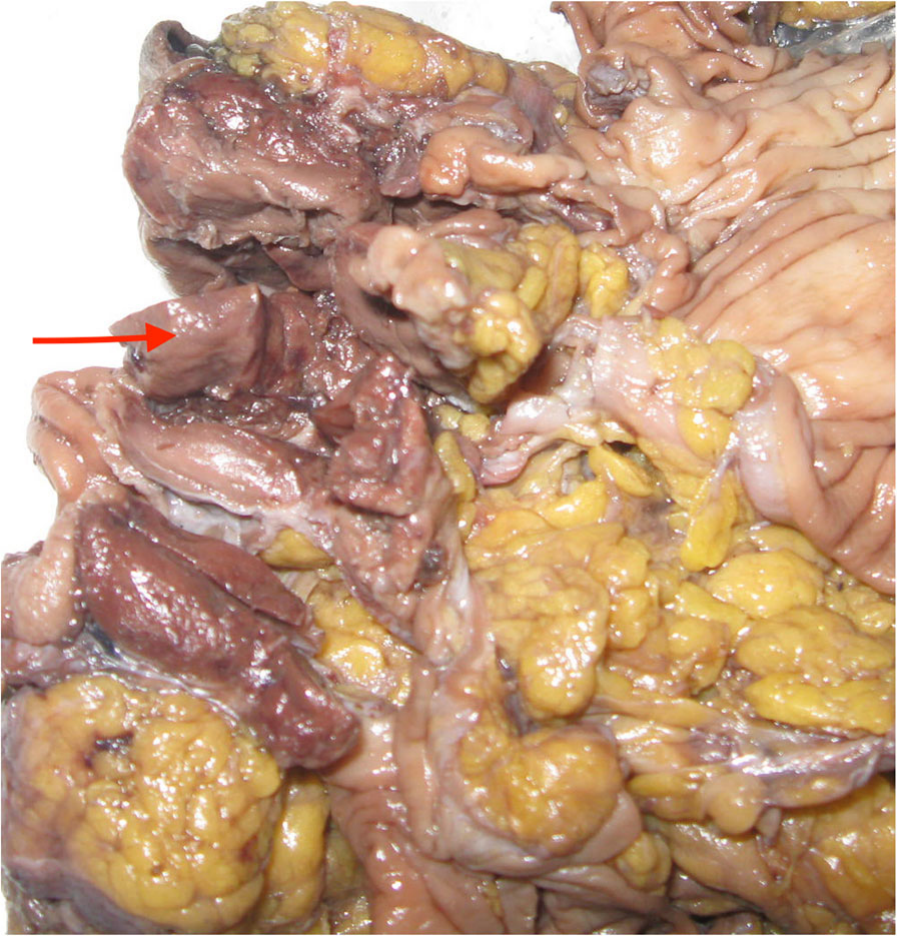
This neoplasm was 5.0 cm in maximum dimension, with features resembling normal spleen

**Fig. 5:**
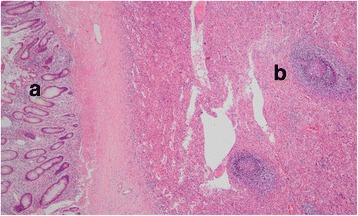
(HE stain, ×10) Splenosis invade into the colon mucosa. **a** Colon mucosa. **b** Splenic nodule showing white pulp surrounded by red pulp

**Fig. 6:**
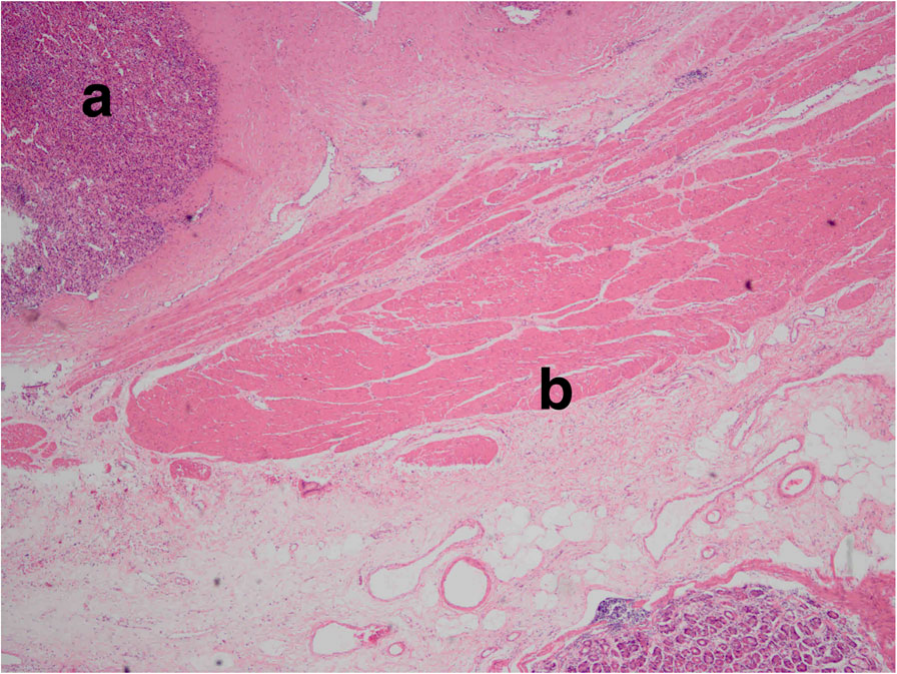
(HE stain, ×10) Splenosis invade into the gastric serosa, near the muscular layer. **a** Gastric mucosa. **b** Gasric muscular layer

The patient underwent laparotomy through a rectus incision of the left lower abdomen. One mass measuring 5 cm × 4 cm × 5 cm (Fig. 4), located between the colon and stomach, was identified. The lesion invaded into the transverse colon and the greater gastric curvature. Local colectomy excision and local stomach excision were performed. Pathological examination revealed this neoplasm to be 5.0 cm in maximum dimension with features resembling those of a normal spleen (Fig. 5). The diagnosis was amended to splenosis invading the colon mucosa and gastric serosa near the muscular layer (Fig. 6).

## Discussion

The term “splenosis” is used to describe the heterotopic transplantation of splenic tissue following splenic trauma or splenectomy. The term was first used by Buchbinder and Lipkopf in 1939 [1]. Splenosis can be identified in nearly 65% of patients who experience splenic trauma and undergo splenectomy [6].

An accessory spleen is an important differential diagnosis of splenosis. An accessory spleen is a congenital phenomenon, and the accessory spleen is typically located at or near the splenic hilum (80%) or pancreatic tail (17%) [7]. The accessory spleen is supplied by the splenic artery. In contrast to accessory spleen, splenosis, which arises as a result of splenic trauma and splenectomy, derives its blood supply from the surrounding arteries [8]. An accessory spleen may also be identified in post-splenectomy patients [9]. Splenosis can be caused by implantation of detached splenic cells, which may arise from splenic trauma and splenectomy with resultant rupture of the splenic capsule. Implantation of the splenic cells can occur anywhere in the peritoneal cavity or in extraperitoneal locations. The local dissemination and mechanical seeding of the peritoneal cavity by the separated splenic cells is the primary cause of splenosis. One case of haematogenous seeding has been reported in a patient with intracerebral splenosis [10].

GISTs, which arise from the interstitial cells of Cajal, are common submucosal tumours of the gastrointestinal tract and tend to arise in the stomach and small intestine and less frequently in the colon. Activating KIT mutations or platelet-derived growth factor receptor alpha (PDGFRA) mutations play an important role in the development of GISTs [11, 12]. GISTs are fragile tumours. If preoperative therapy is not required, biopsy is not necessary. For most patients, circular protrusions are identified in the gastrointestinal tract by gastrointestinal endoscopy, and submucosal tumours are diagnosed by endoscopic ultrasonography [13]. CT shows a morphological rule mass with Hounsfield unit of 38–79. Digestive bleeding is also reported in some patients. Surgery is the primary therapy of choice for patients with localized GISTs. In our case, a huge mass was showed in CT and digestive bleeding was present. So, clinical diagnosis of GIST was confirmed and surgical resection was performed.

Because most patients with splenosis are asymptomatic, splenosis is often identified incidentally by CT or MRI. It is very difficult to distinguish splenosis from malignancy using these imaging modalities. CT-guided biopsy is an invasive method for the diagnosis of splenosis. Nuclear scintigraphy is a sensitive, non-invasive method to trace ectopic splenic tissue. Scintigraphic agents, such as Tc-99m heat-damaged erythrocytes, can detect small splenic nodules that may be missed by CT [8]. Ferumoxide-enhanced MRI has also been reportedly used for the detection of splenic tumours. Ferumoxide is taken up by reticuloendothelial cells, causing a brief increase in T2 signal intensity followed by a characteristic decrease [14]. These non-invasive methods can improve the likelihood of accurately diagnosing splenosis.

Most cases of splenosis have no clinical significance and do not require surgical treatment. Some cases present clinically with intestinal obstruction, haemorrhage, abdominal pain or haemoptysis [15]. In such cases, surgical intervention is necessary. In our patient, gastrointestinal haemorrhage was the chief problem. During the workup, colonoscopy revealed a large round neoplasm in the splenic flexure of the colon. Whether or not the mass was benign, surgical exploration was necessary. Interestingly, this splenosis was located between the colon and stomach. It is possible that separated splenic cells simultaneously implanted into the ruptured serosa of the colon and stomach 10 years ago.

In general, splenosis should be included in the differential diagnosis for patients with histories of splenic trauma and splenectomy.

## Conclusions

Clinicians should be mindful of the possibility of splenosis in cases of asymptomatic masses in the peritoneal cavity, particularly in patients with histories of splenic trauma and splenectomy. Splenosis must be excluded prior to surgery. Nuclear scintigraphy with 99mTc-sulphur colloid or Tc-tagged heat-damaged red blood cells and ferumoxide-enhanced MRI are non-invasive methods to establish a definitive diagnosis of splenosis to prevent patients from undergoing unnecessary surgery. For symptomatic splenosis, surgical intervention is necessary.

## Abbreviations

CT: Computerized tomography
GIST: Gastrointestinal stromal tumour
MRI: Magnetic resonance imaging
